# Identification and Expression Analysis of a Putative Fatty Acidbinding Protein Gene in the Asian Honeybee, *Apis cerana cerana*

**DOI:** 10.1673/031.013.10101

**Published:** 2013-10-05

**Authors:** Xiaoli Yu, Mingjiang Kang, Li Liu, Xingqi Guo, Baohua Xu

**Affiliations:** 1 State Key Laboratory of Crop Biology, College of Life Sciences, Shandong Agricultural University, Taian, Shandong, 271018, P. R. China; 2 College of Animal Science and Technology, Shandong Agricultural University, Taian, Shandong, 271018, P. R. China

**Keywords:** cloning, real-time RT-PCR

## Abstract

Fatty acid-binding proteins (FABPs) play pivotal roles in cellular signaling, gene transcription, and lipid metabolism in vertebrates and invertebrates. In this study, a putative *FABP* gene, referred to as *AccFABP*, was isolated from the Asian honeybee, *Apis cerana cerana* Fabricius (Hymenoptera: Apidae). The full-length cDNA consisted of 725 bp, and encoded a protein of 204 amino acids. Homology and phylogenetic analysis indicated that *AccFABP* was a member of the FABP multifamily. The genomic structure of this gene, which was common among FABP multifamily members, spanned 1,900 bp, and included four exons and three introns. Gene expression analysis revealed that *AccFABP* was highly expressed in the dark-pigmented phase of pupal development, with peak expression observed in the fat bodies of the dark-pigmented phase pupae. The *AccFABP* transcripts in the fat body were upregulated by exposure to dietary fatty acids such as conjugated linoleic acid, docosahexaenoic acid, and arachidonic acid. Transcription factor binding sites for Caudal-Related Homeobox and functional CCAAT/enhancer binding site, which were respectively associated with tissue expression and lipid metabolism, were detected in the 5′ promoter sequence. The evidence provided in the present study suggests that *AccFABP* may regulate insect growth and development, and lipid metabolism.

## Introduction

Fatty acid-binding proteins (FABPs) are abundant intracellular proteins that bind longchain fatty acids (FAs) with high affinity, and belong to a superfamily of hydrophobic, ligand-binding proteins that are expressed widely in vertebrates and invertebrates (Wang et al. 2009). The physiological roles played by FABPs include, but are not limited to, the intracellular trafficking of FAs, lipid metabolism, growth and differentiation, gene transcription, and cytoprotection (Veerkamp and Maatman 1995; Glatz and Van der Vusse 1996; Storch et al. 1996). The FABP family forms a group of at least 12 distinct proteins, as well as the cellular retinoid-binding proteins. These proteins are often named on the basis of the tissue in which each member was first identified in vertebrates (Hertzel and Bernlohr 2000; Liu et al. 2008). Although showing low similarity among the amino acid sequences, the reported FABP structures have similar tertiary structures, involving a β-barrel within which the ligand-binding cavity is located (Marcelino et al. 2006). Moreover, the coding sequence is always interrupted by three introns of varying sizes. These introns are inserted in analogous positions, suggesting that they are of ancient evolutionary origin (Schaap et al. 2002).

Numerous reviews have focused on the structural features and functions of the FABP family (Stewart 2000). For example, FABP1, which is a liver-type FABP, functions in FA uptake and metabolic pathway allocation in vertebrates (Newberry et al. 2003; Storch and Corsico 2008), whereas FABP4, which is an adipocyte-type FABP, affects lipid metabolism and the regulation of gene expression in humans and chickens (Hancke et al. 2010; Shi et al. 2010). Although much research has concentrated on the vertebrate FABPs, there are some previous studies on the functions of insect FABPs. Since the isolation of the first insect FABP from *Schistocerca* *gregaria* in 1990, the number of FABPs identified in insects has been increasing (Esteves and Ehrlich 2006). FABPs from *Apis* *mellifera* may be associated with the regulation of caste differentiation, and their expression could be nutritionally regulated (Evans and Wheeler 1999). The detection of FABPs in the nuclei of *S. gregaria* cells showed that there was a link between signal transduction and gene expression (Haunerland et al. 1993). However, recent studies on the gene expression and function of FABPs in insects are limited.

FABPs are currently thought to bind saturated and unsaturated long-chain FAs, play essential roles in cellular FA transport and utilization, and be indirectly involved in the FA-mediated regulation of gene expression. FABPs increase FA solubility, and facilitate the transport of FAs from the plasma membrane to either sites of FA oxidation or the nucleus, possibly for regulatory functions (Zimmerman and Veerkamp 2002; Storch and Corsico 2008). FAs are utilized as an energy source in various functions, and act as intracellular signaling molecules. In addition, FAs play a role in the transcription of genes that encode proteins involved in lipid metabolism (Duplus et al. 2000). Conjugated linoleic acid (CLA), which is a type of FA, has unique effects on lipid metabolism, and also provides several health benefits for humans and insects (Park et al. 2006). Docosahexaenoic acid (DHA) and arachidonic acid (AA), which are major FAs of the retina, affect gene expression by regulating the activity and concentration of transcription factors within the nucleus (Saino-Saito et al. 2009).

The Asian honeybee, *A. cerana cerana* Fabricius (Hymoneptera: Apocrita), is an important beneficial insect in agriculture. It is widely farmed in China, and is a fundamental and valuable model system for many studies. This study determined whether the Chinese honeybee had a *FABP* gene, and then identified and characterized this gene for the first time. The honeybees were exposed to dietary FAs, including CLA, DHA, and AA, and the potential role of this gene in lipid metabolism was analyzed using real-time RTPCR (qRT-PCR). This study will be a useful reference for the role of FABPs in the honeybee, and help in the breeding of honeybees.

## Materials and Methods

### Experimental design

*A. cerana cerana* were maintained at Shandong Agricultural University, China. The entire bodies of second (L2), fourth (L4), and fifth (L5) larval instars, and early (Pw), pink (Pp), and dark-pigmented (Pbd) phase pupae were taken from the hive. The adult workers (20 days and 50 days after emergence) were collected at the entrance of the hive when returning to the colony after foraging. The larvae and pupae were collected and staged according to the criteria of Michelette and Soares (1993). The adults were obtained by paint marking newly emerged bees (1 day old), and then collecting them after 20 days and 50 days. The 20-day-old adults, which were used for further studies, were laid in boxes that were made in o laboratory, and the boxes were placed into incubators that were kept at a constant temperature (32° C) and humidity (70%) (Alaux et al. 2010).

### Sampling of *A. cerana cerana* in the laboratory

Four groups (n = 40/group) of 20-day-old adults were placed in separate boxes and fed a basic adult diet (BAD) containing 30% honey from the source colonies, 70% powdered sugar and water in laboratory. The next day, adults in groups 1–3 were fed with mixtures of BAD and CLA, DHA, and AA, respectively. Within each group, subgroups of adults were fed diets that contained different concentrations of the FAs, which were reported as the concentration gradient in our experiment (Table 1). The CLA was purchased from Qingdao Auhai Biotech Co., Ltd. (Shandong, China). The DHA and AA were purchased from WuHan Hua Yi Da Technology Co., Ltd. (Hu Bei, China). The control bees (group 4) were left untreated. The fat bodies of the adult bees were harvested at the appropriate times for each experiment. Furthermore, samples of brain, muscle, fat body, and epidermal tissue were isolated from Pbd phase pupae. The samples from the different developmental stages and the isolated tissues were immediately stored at - 80° C until nucleic acid extraction. All of the experiments were performed in triplicate.

### Nucleic acid extraction and cDNA synthesis

Total RNA was extracted using Trizol reagent (Invitrogen, Carlsbad, CA, USA, www.invitrogen.com) according to the manufacturer's protocol, and kept at -80° C for later use. To remove potential genomic DNA contamination, the total RNA was digested with RNase-free DNase-I (*Promega*, Madison, WI. USA. http://www.promega.com/). Separately, genomic DNA was isolated using an EasyPure Genomic DNA Extraction Kit in accordance with the manufacturer's instructions (TransGen Biotech, Beijing, China, http://english.transgen.com.cn/). The concentration and quality of RNA and DNA were estimated by agarose gel electrophoresis (Tanon GIS-2010, Tanon Science & Technology Co., Ltd., Shanghai, China, http://www.bio-tanon.com.cn/). Singlestranded cDNA was then synthesized using a reverse transcriptase system (TransGen Biotech) with an adaptor primer oligo d(T)18 at 42° C for 50 minutes.

**Table 1.**
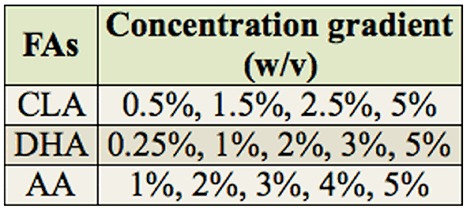
The concentrations of conjugated linoleic acid (CLA), docosahexaenoic acid (DHA), and arachidonic acid (AA) used in the study.

**Table 2.**
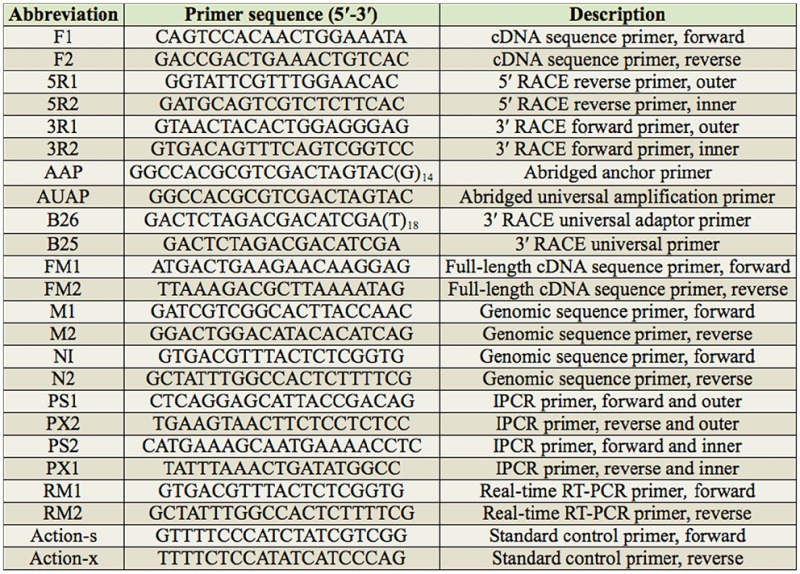
Detailed description of the primers used in the study.

### Amplification of the *AccFABP* gene cDNA fragment

To obtain the internal fragment of *AccFABP*, the primer pair F1 and F2 (Table 2) was designed and synthesized (Shanghai Sangon Biotechnological Company,www.sangon.biogo.net) using the conserved regions of the *F ABP* genes from *A. mellifera*, *Caligus clemensi*, and *Bombus terrestris*. The polymerase chain reaction (PCR) conditions are given in Table 3. All sequencing in the study was performed as follows: the PCR products were purified using a gel extraction kit (TaKaRa, Dalian, China, http://www.takara-bio.com), ligated into the pEasy-T_3_ vector (TransGen Biotech), transformed into *Escherichia coli* strain DH5а, and then sequenced.

### 5′- and 3′-rapid amplification of cDNA ends (RACE) of *AccFABP*

Based on the cloned internal fragments, the specific primer 5R1 and the nested specific primer 5R2 (Table 2) were designed and synthesized for 5′-RACE. First, the DNA Clean-up System (*Promega*, Madison, WI, *USA*) was used to purify the first-strand cDNA, and the 5′-end of the purified cDNA was polyadenylated with deoxycytidine triphosphate by terminal deoxynucleotidyl transferase (TaKaRa). This procedure was followed by ethanol precipitation and resuspension of the DNA in distilled, deionized water. The first round of PCR was performed using primer 5R1, and the universal primer AAP. The PCR product was diluted 20-fold for a second round of amplification using the nested primer 5R2, and nested universal primer AUAP. For 3′-RACE, the primers 3R1 and 3R2 (Table 2) were also designed based on the sequences of the cloned internal fragments. The first round of PCR was performed using 3R1 and B26. The PCR product from this round was then diluted 10-fold for nested PCR, with a second round of amplification using 3R2 and B25. Both the primary PCR and the nested PCR conditions are shown in Table3.

**Table 3.**
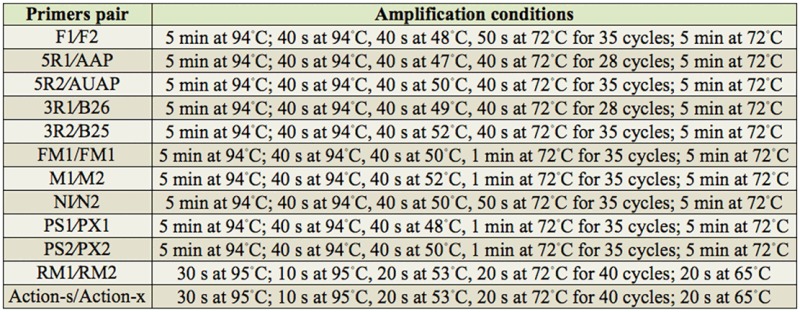
The PCR amplification conditions used in the study.

### Full-length cDNA and genomic sequence amplification of*AccFABP*

By comparing and aligning the above three partial fragments using DNAman software, the full-length cDNA of *AccFABP* was deduced. To verify the integrity and precision of this sequence, PCR was carried out to amplify the full-length cDNA using the primers FM1 and FM2 (Table 2). Moreover, to obtain the genomic sequence of this *AccFABP* gene, four primers (M1/M2 and N1/N2, respectively) were designed and synthesized based on the cDNA sequence. Using *A. cerana cerana* genomic DNA as a template, PCR was used to amplify the genomic sequence of *AccFABP*. Both reactions are shown in Table 3.

### Amplification of the 5′-flanking regions of *AccFABP*

To obtain the 5′-flanking region of *AccFABP*, an inverse polymerase chain reaction was performed using the restriction endonuclease *Vsp*I, and four primers (Table 2) that were designed based on the genomic sequence of *AccFABP*. The first PCR was performed using the primers PS1 and PX1, and the nested PCR was carried out using primers PS2 and PX2. The reaction conditions are presented in Table 3.

### Sequence and general bioinformatic analysis

The nucleotide and deduced amino acid sequences of *AccFABP* were analyzed and compared using the BLAST search programs (http://blast.ncbi.nlm.nih.gov/Blast.cgi). Open reading frames and multiple protein sequence alignments were predicted using DNAman version 5.2.2 (Lynnon Biosoft Company, USA). The phylogenetic tree and molecular evolutionary analysis were performed using MEGA version 4.1. Transcription factor binding sites in the 5′-flanking regions were predicted using the MatInspector database (http://www.cbrc.jp/research/db/TFSEARCH.html).

**Table 4.**
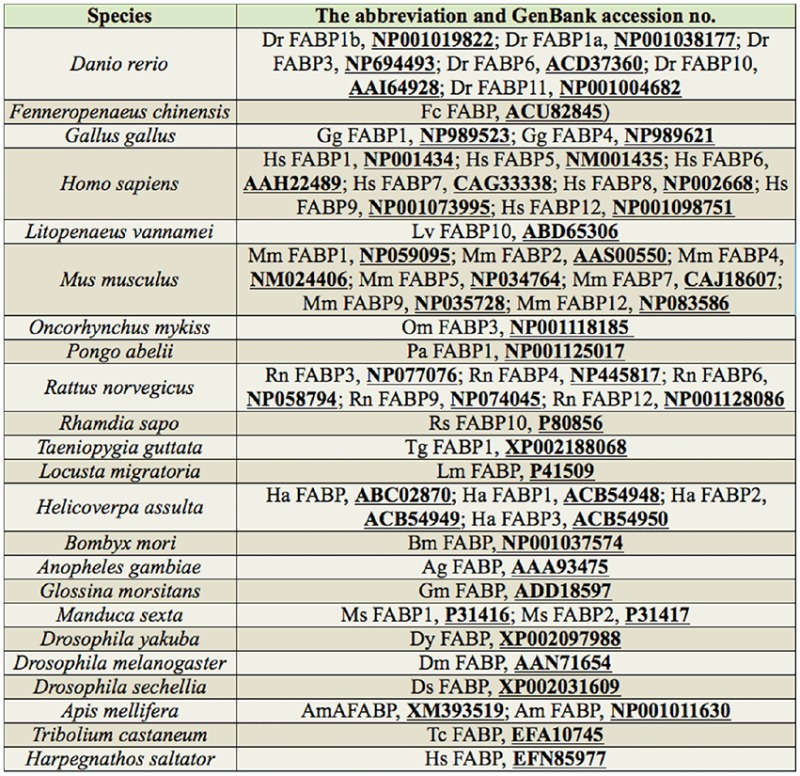
The amino acid sequences used in the evolutionary tree in the study.

### SYBR Green qRT-PCR analysis

Real-time RT-PCR was performed using the CFX 96™ Real-time System (Bio-Rad, USA) according to the manufacturer's instructions. The PCR conditions and primers are presented in Tables 2 and 3. *A. cerana cerana β-actin* (GenBank accession no. XM640276), which is a housekeeping gene, was used for normalization. The samples were run in triplicate, and the expression level of the *AccFABP* transcript relative to *β-actin* was calculated using the 2^-ΔΔCt^ comparative CT method (Livak et al. 2001). The data were interpreted as triplicate mean ± SE (standard error), and presented as the n-fold difference relative to *β-actin*. All the data were analyzed by one-way ANOVA analysis and post-hoc Tukey test using Statistical Analysis System (SAS) version 9.1 (Version 8e, SAS Institute, Cary, NC, USA, http://www.sas.com). Significance was set at *p* < 0.05.

## Results

**Figure 1.**
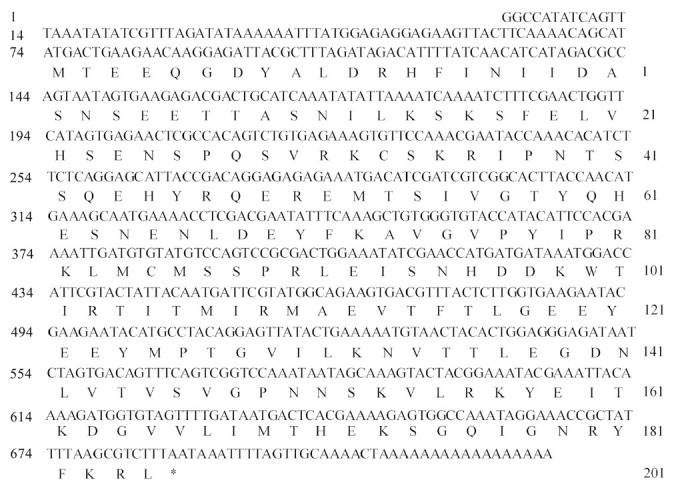
The nucleotide and deduced amino acid sequences of *AccFABP*. Nucleotide numbering begins at the first bp at the 5′ end. Amino acid numbering begins at the first methionine. The stop codon is marked by an asterisk. The sequence has been deposited into GenBank (GenBank accession number: HQ828078). High quality figures are available online.

**Figure 2.**
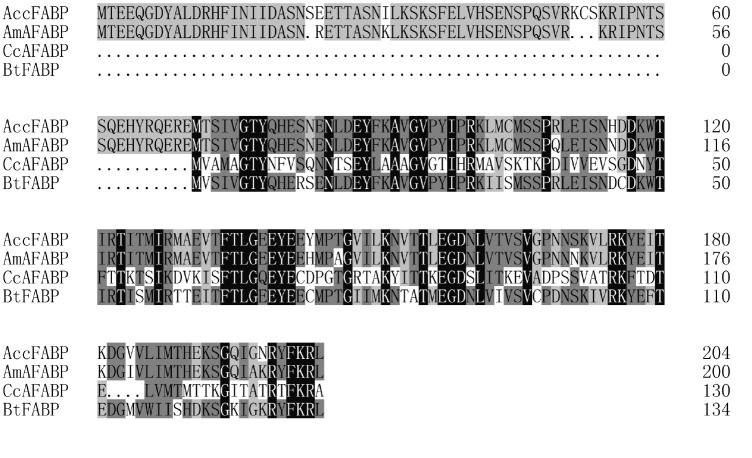
Amino acid sequence alignment of FABPs. The alignment includes the following sequences: *Apis mellifera* (AmAFABP, XM393519); *Caligus clemensi* (CcAFABP, ACO 14989); *Bombus terrestris* (BtFABP, XP003397965). Identical amino acid residues in this alignment are shaded in black and gray. High quality figures are available online.

**Cloning and sequence analysis of *AccFABP*** The full length cDNA of the putative FABP, which was named *AccFABP*, was isolated from the Chinese honeybee (GenBank accession no. HQ828078). The cDNA sequence was 725 bp in length, containing a 73-bp 5′-untranslated region and a 37-bp 3′-untranslated region (Figure 1). The 615-bp open reading frame encoded a protein of 204 amino acids with a predicted molecular weight of 23.45 kDa, and an estimated isoelectric point of 5.91. Analysis of the *AccFABP* cDNA sequence revealed that it appeared to be most similar to the FABP gene sequences included in the National Center for Biotechnology Information database (NCBI, http://www.ncbi.nlm.nih.gov/Blast/). The deduced amino acid sequence of AccFABP was aligned with the corresponding sequences of AmAFABP from *A. mellifera* (93% identity), BtFABP from *B. terrestris* (50% identity), and CcAFABP from *C. clemensi* (24% identity) (Figure 2).

### Evolutionary analysis of FABPs

**Figure 3.**
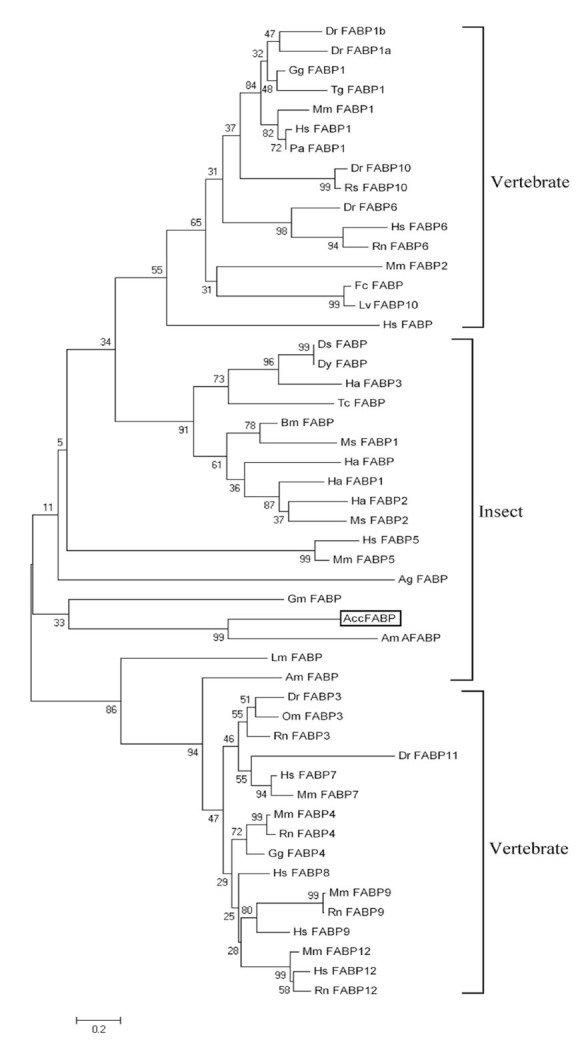
The phylogenetic relationship of AccFABP to other FABPs in insects and vertebrates using neighbor-joining distance analysis. The numbers at each node mark the confidence level of the posterior probability. The amino acid sequence abbreviations are presented in Supplemental Table 4. High quality figures are available online.

To determine the phylogenetic position of AccFABP, an evolutionary tree was constructed based on the amino acid sequences of reported FABPs using MEGA 4.1 software by the neighbor-joining method (Figure 3). According to the phylogenetic tree, the insect FABP family, including AccFABP, was unambiguously separated from the FABP family in vertebrates, excluding Hs FABP and Mm FABP5, which are from human and mouse, respectively. Within the insect portion of the tree, AccFABP and AmAFABP were also distinctly segregated from the FABPs of other insects. This phylogenetic analysis has highlighted the molecular relationships between the members of the FABP family and their evolution.

### Genomic structure analysis of *AccFABP*

**Figure 4.**
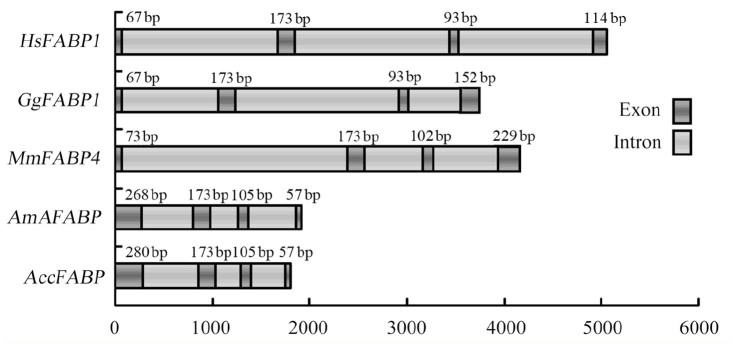
A schematic representation of the DNA structures of various FABPs. The DNA sequences are from *Homo sapiens* (Hs FABPI, NC000002), *Gallus gallus* (Gg FABP I, NC006091), Mus *musculus* (Mm FABP4, NC000069) and *Apis mellifera* (AmAFABP, NC007070). The exons are highlighted with black bars, and the introns are indicated with gray bars. The lengths of the exons are indicated by the number of bases. High quality figures are available online.

To further elucidate the properties of *AccFABP*, the genomic sequence was determined. The genomic sequence of *AccFABP* (GenBank accession no. HQ828080) spanned 1,900 bp, and included four exons and three introns. All of the introns of *AccFABP* possessed the typical features of introns, such as being AT-rich, and being flanked by 5′ splice donor GT and the 3′ splice acceptor AG signals. Comparison of the genomic organization of the different FABP genes indicated that they all possessed the same number and position of exons and introns, but that the intron length was variable (Figure 4). This conservation of genomic structure between its members is one reason that the FABPs are classified as a multigene family.

### Identification of putative transcription factor binding sites in the 5′-flanking region of *AccFABP*

**Figure 5.**
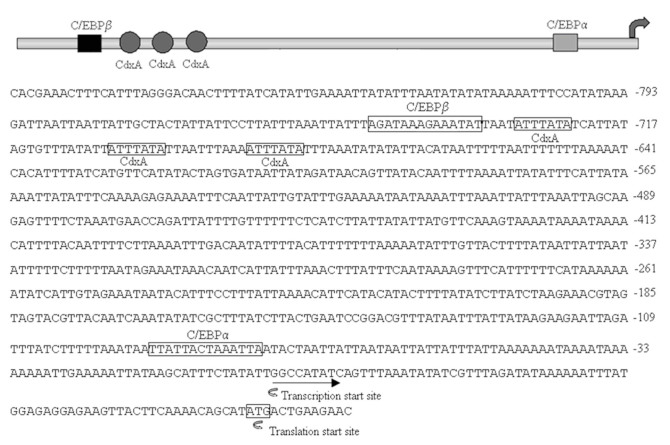
The nucleotide sequences and putative transcription factor binding sites of the 5′-flanking region of *AccFABP*. The translation start site ATG and the transcription start site are marked with arrowheads. The transcription factor binding sites are indicated with boxes. High quality figures are available online.

To understand the mechanism involved in the expression and regulation of this gene, inverse PCR was used to amplify the 941-bp DNA fragment upstream of the *AccFABP* translation start site. Several transcription factor binding sites in the 5′ promoter region that could influence transcription were predicted (Figure 5). Predicted binding sites for Caudal-Related Homeobox, which contributed to tissue-selective expression (Ericsson et al. 2006), were found in the 5′-flanking region of *AccFABP*. In addition, functional CCAAT/enhancer-binding sites (C/EBPα,C/EBPβ), which are thought to play a central role in the regulation of intermediary metabolism (Bernlohr et al. 1997), were also found in the promoter sequence of *AccFABP*.

### Expression profiles of *AccFABP* in different developmental stages and tissues

**Figure 6.**
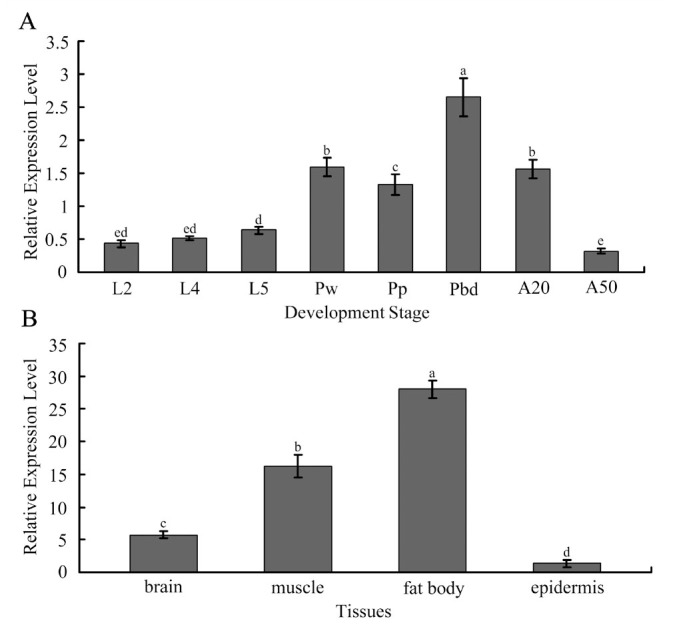
The expression profiles of *AccFABP* as determined by real time RT-PCR. A: The expression in the entire bodies of 2nd (L2), 4th (L4), and 5th (L5) larval instars; early (Pw), pink (Pp) and dark-pigmented (Pbd) phase pupae; and adult workers (20 d and 50 d after emergence). B: The distribution in the brain, muscle, fat body and epidermis. The *β-actin* gene is used as a standard to allow normalization of the amount of template in the PCR reactions. The bars represent the triplicate mean ± SE from three individuals (n = 3). The letters above the bars indicate significant differences as determined using SAS software analysis (*p* < 0.05). High quality figures are available online.

The expression profiles of *AccFABP* in different developmental stages and tissues were determined by qRT-PCR. As shown in Figure 6A, in the larval stage, the expression levels of *AccFABP* increased slightly but showed no significant differences during the successive instars (L2, L4, and L5). However, the transcription rates of *AccFABP* increased more dramatically during the successive pupal stages (Pw, Pp, and Pbd), and the transcript accumulation of *AccFABP* peaked at the Pbd stage. In adults (20- and 50-day-old), *AccFABP* expression decreased gradually, and reached a basal level in the 50-day-old adults.

Because *AccFABP* was highly expressed at the Pbd stage, the tissue-specific expression of *AccFABP* was analyzed at this stage. As shown in Figure 6B, the expression levels were normalized against the levels in the fat body. The transcript levels of *AccFABP* in thefat body were 5.02-fold higher than in the brain and 1.27-fold higher than in the muscle. Thus, *AccFABP* exhibits a tissue-specific pattern of expression (p < 0.05).

### Expression pattern of *AccFABP* in response to dietary CLA, DHA, and AA

**Figure 7.**
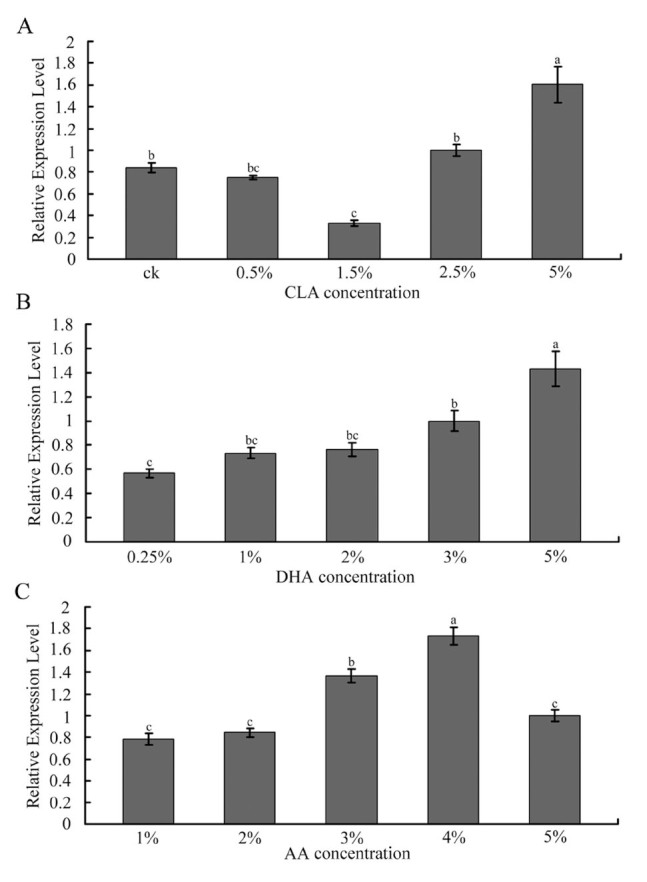
The expression of *AccFABP* in response to dietary conjugated linoleic acid (CLA), docosahexaenoic acid (DHA) and arachidonic acid (AA). The expression analysis was performed using total RNA extracted from 20-day-old adult bees at different times after treatment with CLA (A), DHA (B) and AA (C). Ck indicates the control group. The *β-actin* gene is used as a standard to allow normalization of the amount of template in the PCR reactions. The bars represent the triplicate mean±SE from three individuals (n = 3). The letters above the bars indicate significant differences as determined using SAS software analysis (*p* < 0.05). High quality figures are available online.

To investigate the effects imposed by F As, qRT-PCR was used to determine the expression level of fat body *AccFABP* in response to dietary administration of CLA, DHA, and AA. As shown in Figure 7, dietary CLA at low concentrations reduced the expression of *AccFABP* compared with the controls, and the expression levels of *AccFABP* increased gradually from the low level observed in the 1.5% CLA group, and to a peak in the 5% CLA group. Dietary DHA and AA both upregulated the transcription levels of *AccFABP*, with peak transcription occurring in the 5% DHA group and the *4%* AA group, respectively.

## Discussion

FABPs possess comprehensive and interdependent functions in the regulation of gene expression and intracellular FA transport (Zimmerman and Veerkamp 2002). Many studies have focused on the structure, expression and function of vertebrate and invertebrate FABPs. However, these proteins remain largely unstudied in many insect species. The current study reports the identification and characterization of a putative FABP gene in *A. cerana cerana* for the first time and presents evidence for its potential roles in lipid metabolism.

The cDNA sequence of this putative *AccFABP* gene encoded a protein of 204 amino acids. Protein alignment revealed that there was high identity between AccFABP and other insect FABPs. Phylogenetic analysis showed that AccFABP was contained within the insect group, but it was clearly separated from the FABPs of other insects, except for AmAFABP. Moreover, a comparison of the genomic organization of the *FABP* genes revealed that all insect *FABP* genes were organized in four exons and three introns, and that the introns are located in conserved positions, although they vary in size. Taken together, we hypothesize that *AccFABP* is one of the FABP superfamily members, and we suggest a phylogenetic and functional relationship with other reported FABPs.

The regulation of the FABP expression in insects has not been studied extensively. However, some work on promoter characterization and expression studies has been done to explore FABPs. In the present study, the expression profiles of *AccFABP* in different developmental stages and tissues were investigated by using qRT-PCR. *AccFABP* was highly expressed in the Pbd pupae and the adult prophase. It also displayed specific expression in the fat bodies of Pbd pupae. Moreover, the predicted transcription-factor binding sites for CaudalRelated Homeobox in the promoter region of *AccFABP* may contribute to the tissueselective expression of *AccFABP* in the fat body (Ericsson et al. 2006). It is proposed that worker-destined larvae begin receiving food that includes F As as an energy source, and that the lipid metabolism matures along with the development of the bees. Lipid metabolism is weak in aged bees. The fat body in insects is generally regarded as a major lipid storage organ. Furthermore, the fat body is involved in various humoral functions, including nutrition, reproduction, and longevity (Ottaviani et al. 2011). The transport of FAs from the fat body to other tissues is an aspect of lipid metabolism that is essential for insect development andmetamorphosis, and this transport is determined by developmental stage, nutritional state, sex, and migratory flight (Arrese et al. 2006; Liu et al. 2009). Taken together, these observations suggest that *AccFABP* may be associated with the regulation of tissue-specific gene expression and lipid metabolism during honeybee development.

To further elucidate the function of *AccFABP* in lipid metabolism, we exposed honeybees to dietary CLA, DHA, and AA. These three FAs, which are required nutritional elements, upregulated the expression of *AccFABP* at the level of transcription initiation. Furthermore, the C/EBP-binding sites predicted in the 5′-flanking region of *AccFABP* may be involved in the regulation of FABP genes and intermediary metabolism (Bernlohr et al. 1997). Recent studies have shown that CLA accumulated in the tissues of houseflies and silkworms after breeding, affecting lipid metabolism (Park et al. 2000, 2006). The levels and ratio of DHA and AA could influence development across animal species, and dietary deficiency could cause reproductive failure and reduced growth (Davis-Bruno et al. 2011). The different expression patterns observed after treatment with dietary CLA, DHA, and AA suggest that *AccFABP* may be involved in the lipid metabolism of the fat body, which is essential for honeybee growth and development. This study also provides a useful reference about the nutrition and breeding of the Chinese honeybee. However, further transgenic analysis and elucidation of the involved mechanisms are necessary.

## Abbreviations:

AA,: arachidonic acid
C/EBP,: functional CCAAT/enhancer-binding site
CLA,: conjugated linoleic acid
DHA: docosahexaenoic acid
FA,: fatty acids
FABP,: fatty acid-binding protein
Pbd,: dark-pigmented phase
**qRT****PCR,**: real-time reverse transcription PCR
